# Computational design of the temperature optimum of an enzyme reaction

**DOI:** 10.1126/sciadv.adi0963

**Published:** 2023-06-28

**Authors:** Florian van der Ent, Susann Skagseth, Bjarte A. Lund, Jaka Sočan, Julia J. Griese, Bjørn O. Brandsdal, Johan Åqvist

**Affiliations:** ^1^ Department of Cell and Molecular Biology, Uppsala University, Biomedical Center, Box 596, SE-751 24 Uppsala, Sweden.; ^2^ Hylleraas Centre for Quantum Molecular Sciences, Department of Chemistry, University of Tromsø–The Arctic University of Norway, N9037 Tromsø, Norway.; ^3^ National Institute of Chemistry, SI-1001 Ljubljana, Slovenia.

## Abstract

Cold-adapted enzymes are characterized both by a higher catalytic activity at low temperatures and by having their temperature optimum down-shifted, compared to mesophilic orthologs. In several cases, the optimum does not coincide with the onset of protein melting but reflects some other type of inactivation. In the psychrophilic α-amylase from an Antarctic bacterium, the inactivation is thought to originate from a specific enzyme-substrate interaction that breaks around room temperature. Here, we report a computational redesign of this enzyme aimed at shifting its temperature optimum upward. A set of mutations designed to stabilize the enzyme-substrate interaction were predicted by computer simulations of the catalytic reaction at different temperatures. The predictions were verified by kinetic experiments and crystal structures of the redesigned α-amylase, showing that the temperature optimum is indeed markedly shifted upward and that the critical surface loop controlling the temperature dependence approaches the target conformation observed in a mesophilic ortholog.

## Teaser

Computer simulations of variants of a cold-adapted enzyme are used to design the temperature optimum of the catalytic reaction.

## INTRODUCTION

The fact that a number of cold-adapted enzymes form psychrophilic species show an anomalous temperature optimum, which is not related to protein unfolding, has attracted much attention (*1*–*9*). It has been suggested that the phenomenon could be due to the active site being more heat-labile than the rest of the enzyme (*1*), which would correspond to a local or partial melting rather than global unfolding as captured by calorimetric techniques or fluorescence measurements. Another explanation brought forward invokes the concept of a negative heat capacity difference between the transition state and the reactant state of the reaction (*5*, *6*). This would cause a curvature in the plot of activation free energy versus temperature and a corresponding rate optimum, due to nonconstant activation enthalpy and entropy components.

The α-amylase from the psychrophilic bacterium *Pseudoalteromonas haloplanktis* (AHA) is probably the most thoroughly studied cold-adapted enzyme that shows such an anomalous *T*-optimum, which, in this case, is about 15°C lower than the melting temperature (*T*_m_) (*1*). This enzyme has 47% sequence identity with the best characterized mesophilic ortholog, namely, the porcine pancreatic α-amylase (PPA) that also has a very similar three-dimensional (3D) structure (*10*, *11*). AHA is about threefold faster than the porcine enzyme at 10°C with the 4-nitrophenyl-α-d-maltoheptaoside-4,6-*O*-ethylidene substrate but reaches its maximum rate already at around room temperature (*1*). Earlier computer simulations of the catalytic reactions of AHA and PPA, based on molecular dynamics (MD) free energy calculations using the empirical valence bond (EVB) method (*12*, *13*), could directly reproduce the *T*-optimum of AHA and explain its origin (*7*). It was found that a particular binding interaction, between Asp^264^ of the enzyme and the 2′- and 3′-hydroxyl groups at the −1 position of the oligosaccharide substrate, starts to break precisely at room temperature. This gives rise to an off-pathway inhibitory thermodynamic state along the reaction path whose population becomes dominating at higher *T*, thus causing a decline of the catalytic rate (*7*, *8*). In the porcine enzyme, on the other hand, the interaction was found to be stable up to temperatures approaching *T*_m_ (*8*). Hence, in the case of AHA, the explanation for the optimum could perhaps be considered as “local melting,” which causes a distinct break in the Arrhenius plot and a concomitant *T*-optimum. It should also be noted here that Asp^264^ is totally conserved (together with the two catalytic residues Asp^174^ and Glu^200^) in the large family of enzymes to which the α-amylases belong (*14*, *15*). It is thus likely that inactivation due to breakage of the Asp^264^-substrate interaction is a common phenomenon in many of these enzymes.

**Fig. 1.**
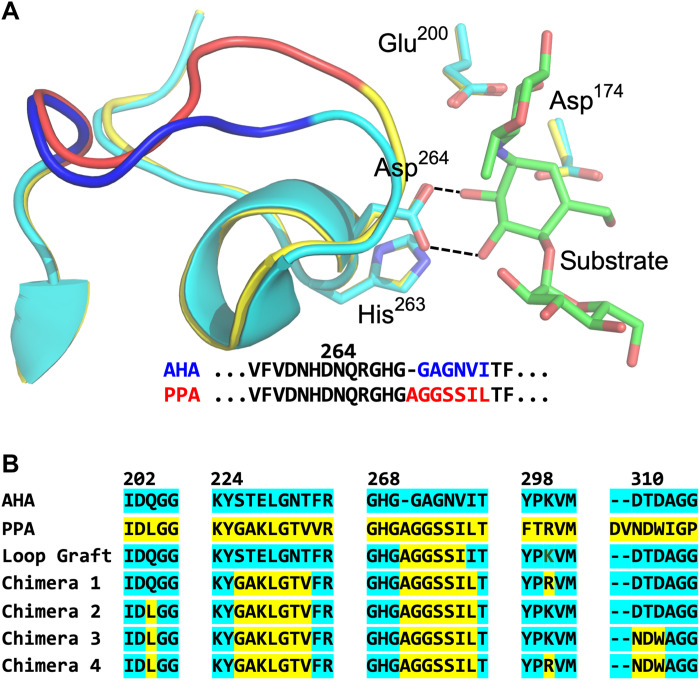
Structures of the β7-α7 loop and sequence regions selected for mutagenesis. (**A**) View of loop region following Asp^264^ in AHA (*10*) (cyan/blue) and PPA (*11*) (yellow/red). The catalytic residues Asp^174^ and Glu^200^ are also shown. (**B**) Sequence regions chosen for mutagenesis in the different chimeric variants.

These findings directly suggest that it should be possible to redesign the cold-adapted enzyme so as to push the optimum toward higher temperatures, if the optimum is basically controlled by a specific substrate binding interaction. The key enzyme amino acid Asp^264^ is located in a loop region between the secondary structure elements β7 and α7 of domain A (Fig. 1A) (*10*, *11*). This conserved side chain has six residues upstream and six downstream that also are identical between the psychrophilic and mesophilic enzymes, which suggests that its conformational behavior may be dictated by nonlocal interactions. The two sequences diverge after Gly^270^, where PPA has an Ala insertion, and the β7-α7 loop adopts different conformations (*10*, *11*), but the two structures converge again at Val/Ile^275^ (Fig. 1A). The MD simulations (*7*) also showed a distinctly higher mobility of the loop in AHA at 25°C compared to PPA. Since only the cold-adapted AHA enzyme showed instability of the Asp^264^-substrate interaction above room temperature, we hypothesized that if its β7-α7 loop conformation could be brought to that found in PPA, then its *T*-optimum could possibly be raised.

It can be noted that computational design of the temperature dependence of enzyme reactions has, as far as we know, never been attempted before. This problem is obviously much more difficult than, e.g., altering protein thermal stability based on computations, where success was reported already in 1998 (*16*). The main reason is that the activation free energy of the reaction must be calculated with reasonable fidelity as a function of temperature, which is not trivial (*13*). In the case of AHA, experimental attempts to reengineer the enzyme have been made based on selection of mutants from multiple sequence alignments (*17*). For example, a variant with seven residues taken from PPA was found to increase *T*_m_ by 8°C. It had *k*_cat_ and *K*_M_ values that were similar to those of PPA (two- and threefold lower than AHA, respectively), but no measurement of the temperature optimum was reported (*17*). In this work, we sought to computationally identify a small set of mutations taken from the porcine sequence that could alter the β7-α7 loop conformation and also increase the *T-*optimum of AHA. The strategy was then: (i) to examine the stability of the Asp^264^-substrate interaction for different sets of mutations at 25°C by plain MD simulations, (ii) to predict the *T*-optimum of the catalytic reaction from MD/EVB simulations (*12*, *13*) at different temperatures for the most promising candidate enzyme, and (iii) to validate the predictions experimentally in terms of steady-state kinetics and structure determination. The results show that such a computational approach to designing enzyme temperature dependence can be effective.

## RESULTS

### Identification of possible sequence regions for mutagenesis

**Fig. 2.**
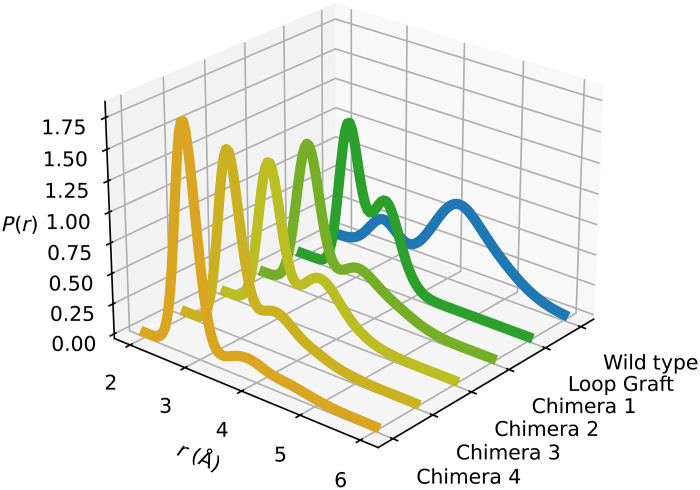
Predicted stability of the Asp^264^-substrate interaction. Calculated probability density for the Asp^264^-substrate oxygen (Oδ2-O2) distance from MD simulations of WT AHA and the different chimeric models. The active reactant (target) state corresponds to the peak at ~2.8 Å.

The goal of the present work was both to confirm the connection between the Asp^264^-substrate interaction and the *T*-optimum and to computationally design a variant of AHA with the optimum moved toward higher temperature. To do this, we made designs that we thought could stabilize the Asp^264^-substrate interaction required for catalysis. Structural models were created using the Modeller program (*18*) and filtered by normalized Discrete Optimized Protein Energy (DOPE) score (*19*). For each designed variant, the six top-scoring models were retained, and MD simulations (at 25°C) were carried out for each model. The behavior of the β7-α7 loop conformation was monitored, and the distribution of active and inactive structures determined. The loop itself was identified as the main target for mutations since it is the movement of this loop that causes a change in conformation of Asp^264^ and the substrate. A first obvious mutant was thus designed where the AGGSSI loop motif from PPA was just grafted into the β7-α7 loop of AHA. The sequence of this Loop Graft model is shown in Fig. 1B and was considered to represent the minimum number of mutations (five) that could possibly change the loop conformation from that observed in the psychrophilic enzyme to that of the mesophilic counterpart. The MD analysis shows that the Loop Graft model indeed is predicted to improve the overall distribution between active and inactive states but does not effectively suppress the latter (Fig. 2).

Consequently, we concluded that a more extensive overhaul of the surrounding of the β7-α7 loop was required, and four additional regions were selected as targets for mutation (Fig. 1B). The first region was the adjacent side of helix α6 (223 to 234), which is in direct contact with the elongated β7-α7 loop of PPA (fig. S1). The second region was the β5-α5 loop (202 to 210) where Gln^204^ is mutated into leucine in PPA, which packs between α6 and the β7-α7 loop. The third selected mutation is in the chloride ion binding site adjacent to Asp^264^, where Lys^300^ was mutated to arginine. Last, part of the long β8-α8 loop region packs on the other side of the β7-α7 loop where it is in contact with Arg^267^ and His^269^. Here, residues 310 to 312 were mutated to match the sequence found in the mesophile, where particularly the T311D substitution makes direct contact with the loop in the PPA structure (fig. S1) (*11*). On the basis of these four additional sequence regions that are close to the β7-α7 loop in 3D space, we constructed four chimeric variants of AHA (Fig. 1B) and examined their distributions of the active and inactive enzyme-substrate conformations by MD simulation. The results clearly show that the inactive state is progressively suppressed as the chimeras include more of the 3D environment of the β7-α7 loop taken from the mesophilic enzyme (Fig. 2). In particular, the final model (Chimera 4) that combines 16 mutations in all of the four identified regions above is predicted to almost entirely recover the stable hydrogen bond interaction between Asp^264^ and the substrate at 25°C, as opposed to the wild-type (WT) enzyme (*7*). This interaction thus stabilizes the active substrate conformation, and in MD simulations of the mesophilic PPA, it was found to persist up to about 50°C (*8*), in agreement with the experimental rate and melting curves (*1*).

### Computational prediction of the temperature dependence of the best chimera

**Fig. 3.**
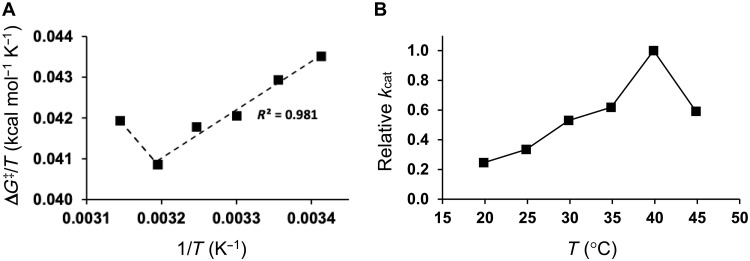
Calculated temperature dependence of the Chimera 4 reaction. (**A**) Computed Arrhenius plot of ∆*G*^‡^/*T* versus 1/*T* from MD/EVB simulations of the glycosylation reaction catalyzed by the structural model of Chimera 4. (**B**) Predicted temperature dependence of the relative rate constant *k*_cat_ for Chimera 4. The SEM for the calculated free energy barriers is 0.19 to 0.32 kcal mol^−1^ (from 120 replicate simulations at each temperature).

The above results thus prompted us to examine the behavior of Chimera 4 in detail by MD/EVB free energy simulations of the catalytic reaction. These calculations were done exactly as reported earlier with a maltopentaose substrate bound to the active site (*7*). Here, 20 independent replicate runs were carried out for each of the six structural models of Chimera 4, and the simulations were repeated at six temperatures between 20° and 45°C. At each temperature, the average activation free energy was calculated which allows the corresponding Arrhenius plot of ∆*G*^⧧^/*T* versus 1/*T* to be constructed (Fig. 3A) (*7*, *13*). It is immediately apparent that there is a break in the Arrhenius plot corresponding to a temperature of ~40°C. The predicted behavior of *k*_cat_ is shown in Fig. 3B, with a rate optimum around this temperature.

This optimum is considerably higher than that obtained from MD/EVB simulations of WT AHA (*7*), indicating that Chimera 4 may be a successful design in shifting the optimum upward. The free energy barrier for the Chimera 4 catalyzed reaction at 25°C is predicted to be ∆*G*^⧧^ = 12.79 ± 0.27 kcal mol^−1^, which is similar to the value calculated earlier for AHA (∆*G*^⧧^ = 13.32 ± 0.08 kcal mol^−1^). Of course, the AHA calculations (*7*) used a more accurate experimental enzyme structure and as many as 300 replicate MD/EVB simulations at each temperature. These differences are probably the main reason for the significantly lower SEM value in that case. The activation enthalpy and entropy for Chimera 4 were also predicted from the low temperature region (20° to 35°C) of the Arrhenius plot in Fig. 3A. The resulting values of ∆*H*^⧧^ = 11.0 and *T*∆*S*^⧧^ = −1.7 kcal mol^−1^ (Table 1) are similar to those reported for PPA with the 4-nitrophenyl-α-d-maltoheptaoside-4,6-*O*-ethylidene substrate between 5° and 25°C, ∆*H*^⧧^ = 11.5 and *T*∆*S*^⧧^ = −2.5 kcal mol^−1^ (with reference temperature *T* = 15°C in both cases) (*17*). In contrast, calculations for the cold-adapted AHA enzyme (*7*) gave ∆*H*^⧧^ = 6.5 and *T*∆*S*^⧧^ = −6.6 kcal mol^−1^, in reasonable agreement with the experimental values (*17*). Hence, Chimera 4 is clearly predicted to be less cold-adapted also in terms of the characteristic activation enthalpy-entropy shift.

**Table 1.** Calculated thermodynamic activation parameters (kcal mol^−1^) for Chimera 4 compared to experimental results for PPA and AHA with the 4-nitrophenyl-α-d-maltoheptaoside-4,6-*O*-ethylidene substrate. The reference temperature for *T*∆*S*^⧧^and ∆*G*^⧧^ is set to 15°C for comparison with the experimental results (*17*). All calculated results are with maltopentaose as the substrate.

	∆*H*^‡^	*T*∆*S*^‡^	∆*G*^‡^
Chimera 4 (calc)	11.0	−1.7	12.7
PPA (expt)*	11.5	−2.5	14.0
PPA (calc)†	10.8	−4.3	15.1
AHA (expt)*	8.3	−5.1	13.4
AHA (calc)†	6.5	−6.6	13.1

*Data from *17*.

†Data from *7*.

### Chimera 4 moves the *T*-optimum upward

**Fig. 4.**
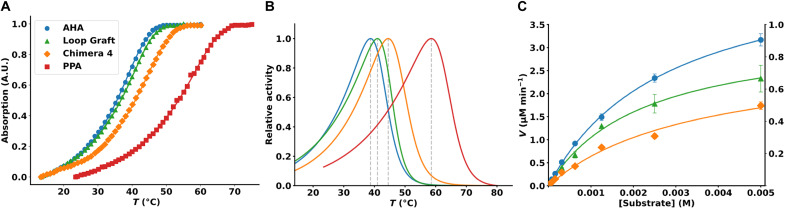
Kinetics of the designed enzyme variants. (**A**) Results from temperature ramping experiments with the 2-chloro-4-nitrophenyl-α-d-maltotrioside substrate for the four enzyme variants AHA (blue), Loop Graft (green), Chimera 4 (orange), and PPA (red). (**B**) Relative *k*_cat_ for the four variants as a function of temperature obtained from the derivatives of the fitted product formation curves in (A). (**C**) Steady-state kinetics measured at 25°C for AHA (blue, left *y* axis), the Loop Graft variant (green, left *y* axis), and Chimera 4 (orange, right *y* axis). Error bars ± 1σ (*n* = 3). A.U., arbitrary units.

In view of these computational results, we decided to measure the effect of two of the designed AHA variants on the temperature dependence of the catalytic reaction with 2-chloro-4-nitrophenyl-α-d-maltotrioside (CNP-G3). This substrate is relatively slowly hydrolyzed and well suited for continuous temperature ramping experiments under steady-state conditions. The Loop Graft variant showed an upward shift of the *T-*optimum of only about 2°C compared to WT AHA (Fig. 4, A and B), which is consistent with its predicted inability to suppress the inactive conformation of enzyme-substrate complex (Fig. 2). Chimera 4, on the other hand, turns out to have a notably larger upshift of the optimum of about 6°C and now shows an activity maximum at around 45°C with this substrate, which is close to that predicted computationally (Fig. 3). The corresponding maximum for the porcine enzyme PPA is found at ~58°C. These results clearly show that the design strategy aimed at stabilizing the β7-α7 loop in its PPA conformation is successful in moving the rate optimum toward higher temperature. It can be noted here that both the WT AHA and PPA optima appear to be slightly higher than reported earlier (*1*), which may be due to the use of a different substrate, but for our purposes, it is the shift between WT AHA and Chimera 4 that is of most interest.

Steady-state kinetics was also measured at 25°C for WT AHA, the Loop Graft model, and Chimera 4 with the CNP-G3 substrate (Fig. 4C). The *k*_cat_ value of 107 ± 2 min^−1^ for WT AHA at this temperature is found be reduced to 73 ± 3 min^−1^ and 18 ± 1 min^−1^, respectively, for the Loop Graft variant and Chimera 4, while the *K*_M_ values are much less affected (table S1). A corresponding *k*_cat_ of 9 min^−1^ has been reported earlier for PPA with CNP-G3 at 25°C (*20*). Hence, it appears that movement of the *T*-optimum upward via changes in the β7-α7 loop region is invariably associated with a loss in activity at lower temperature. However, although Chimera 4 has lost a factor of 6 in activity at 25°C, it is now the fastest variant at 50°C, where it is three times faster than AHA and 1.4 times faster than PPA.

### Crystal structures of Chimera 4

**Fig. 5.**
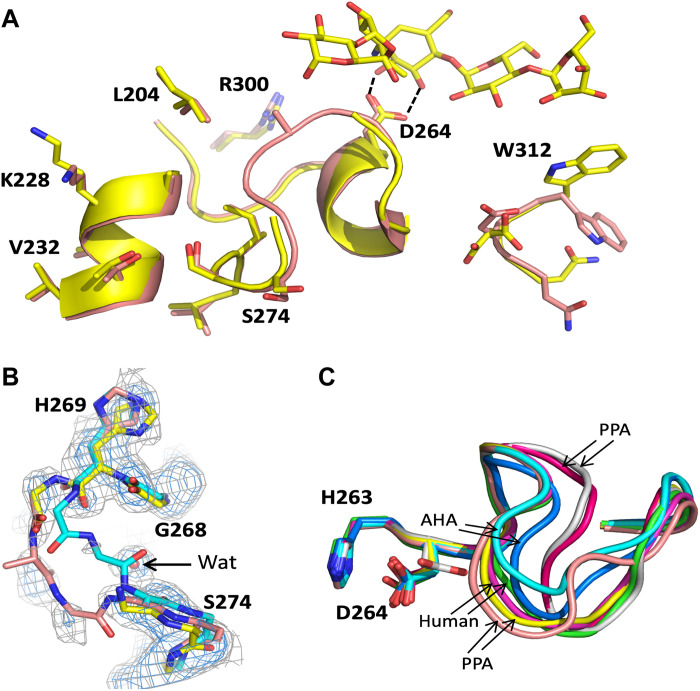
Crystal structure of Chimera 4. (**A**) View of the 2.0-Å resolution structure of the Chimera 4–acarbose complex (yellow) superimposed on the PPA structure 1HX0 (*11*) (pink). The side chains mutated from AHA to Chimera 4 are shown as sticks to illustrate their positions relative to the PPA target structure. Note that the two residues Ala^271^ and Gly^272^ cannot be reliably built for Chimera 4. The key interaction between Asp^264^ and the substrate is also indicated. (**B**) Electron density for the loop regions 268 to 274 where the 1σ level is contoured in blue and the 0.5σ level in gray. The Chimera 4 structure is shown in yellow, PPA (*11*) in pink, and AHA (*10*) in cyan. A water (Wat) molecule present both in the Chimera 4 and PPA structures is shown as a red sphere. (**C**) Superposition of the β7-α7 loop conformations from several crystal structures of AHA: 1G94 (*10*) and 1AQH (*25*); PPA: 1HX0 (*11*), 1UA3 (*22*), 1JFH (*23*), and 1PIF (*24*); and the human enzyme: 1XCX (*21*) and 3BAK (*26*).

To structurally evaluate our computational design, we determined the crystal structures of Chimera 4 in its apo form and in complex with the inhibitor acarbose (Table 2). The inhibitor is known to be rearranged by α-amylases in crystal soaking experiments to form several different products (*21*). The variant observed in the active site here was a pseudo-pentasaccharide which has also been observed in human PPA (*21*). As can be seen in Fig. 5A, the mutated side chains in Chimera 4 are all very close to their position in the corresponding complex with mesophilic PPA. The only exception is the NDW motif (residues 311 to 313) where the side-chain conformations differ somewhat. It appears that the extended β2-α2 and β8-α8 loops in PPA (*11*) that pack against this motif are responsible for these differences, as Chimera 4 retains the shorter loops from AHA.

**Table 2.** X-ray data collection and refinement statistics for the apo and holo forms of Chimera 4. Values in parentheses are for the highest-resolution shell. Five percent of the reflections were used in the *R*_free_ calculations.

Parameter	Chimera 4 apo	Chimera 4–acarbose
**Data collection**		
X-ray source	ID23, ESRF	ID30a, ESRF
Space group	P2_1_2_1_2_1_	P2_1_2_1_2_1_
Unit cell dimension, Å/°	*a* = 68.9, *b* = 80.9, *c* = 128.5 α = 90, β = 90, γ = 90	*a* = 68.6, *b* = 81.1, *c* = 129.5 α = 90, β = 90, γ = 90
Resolution range, Å	68.88–1.74 (1.77–1.74)	38.69–2.05 (2.10–2.05)
Wavelength, Å	0.97625	0.96770
Total reflections	496,926 (25,661)	243,990 (25,013)
No. of unique reflections	74,537 (3,892)	84,789 (8,759)
Multiplicity	6.7 (6.6)	2.9 (2.9)
Completeness, %	99.7 (96.1)	96.7 (99.4)
Mean (〈*I*〉/σ〈*I*〉)	12.8 (0.9)	6.5 (1.1)
R-merge	0.077 (1.718)	0.112 (0.864)
CC_1/2_	0.998 (0.363)	0.993 (0.471)
**Refinement**		
PDB entry no.	8CQG	8CQF
Resolution range, Å	68.48–1.74	38.69–2.05
Number of reflections	74,431	84,778
*R*_work_/*R*_free_, %	17.65/20.51	16.87/19.78
**No. of:**		
Non-H atoms	3,961	3,965
Amino acid residues	447	448
Water molecules	396	297
Ligands	11	10
Ions	3	3
**RMS.deviations:**		
Bond lengths, Å	0.009	0.010
Bond angles, °	0.960	1.200
**Avg. B-factor, Å** ^2^		
All atoms	38.15	41.17
Protein	37.39	39.88
Solvent	43.88	41.38
Ligands	64.76	65.59
		
Ions	31.40	34.79
Ramachandran favored/outliers, %	96.61/0.00	96.17/0.00
Rama *Z*-score	−0.41 ± 0.38	−0.60 ± 0.39
Rotamers favored/outliers, %	93.60/0.53	92.41/0.54
Clashscore	2.03	2.69
MolProbity score	1.19	1.32

The electron density is weak for the two residues Ala^271^ and Gly^272^ of the remodeled β7-α7 loop, in both the apo- and holo-structures, so that their backbone conformation cannot be confidently built. There is, however, clear indication from the partial density that the loop conformation is closer to that of PPA than AHA, as intended for Chimera 4 (Fig. 5B). For example, both PPA and Chimera 4 have a well-defined water position that bridges between the carbonyl oxygens of Asn^265^ and Gly^273^, and this water molecule would clash with the AHA backbone. When analyzing a number of crystal structures (*10*, *11*, *21*–*26*) of PPA, AHA, and the human PPA (87% identical to pig), it becomes evident that the β7-α7 loop can adopt a range of different conformations and thus appears quite flexible (Fig. 5C). Moreover, analysis of the deposited density map for the PPA structure with Protein Data Bank (PDB) code 1UA3 (*22*) shows that the loop there can basically be traced in two alternative ways, corresponding to the 1HX0 (*11*) and 1UA3 conformations (Fig. 5C). An additional complication here turns out to be crystal contacts between neighboring protein molecules and the β7-α7 loop found in several of the analyzed structures, including the inhibitor complexes of AHA (1G94) (*10*) and PPA (1HX0) (*11*) which makes the preferred conformations in the native enzymes less certain. In our apo- and holo-structures of Chimera 4, there are no crystal contacts with the β7-α7 loop region, which may be one reason for the weak density discussed above.

### Mobility of the β7-α7 loop

**Fig. 6.**
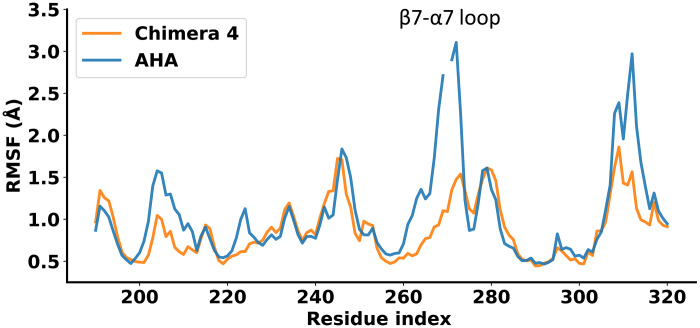
Mobility differences between AHA and Chimera 4. Calculated average backbone positional RMSF per residue, from MD simulations at 25°C in the reactant state of AHA (blue) and Chimera 4 (orange).

It appears that the mobility of the loop downstream of the key residue Asp^264^, which “holds” the substrate in place, is intimately connected to the temperature dependence of the catalytic reaction (*7*). Our earlier computer simulations showed a marked shift in mobility of the β7-α7 loop between PPA and AHA (a factor of ~2), and this was the most pronounced difference in root mean square positional fluctuations (RMSF) for the protein backbone. With the crystal structure of Chimera 4, we can examine by MD simulations of the reactant state how much the mobility of the β7-α7 loop has actually been reduced in our design. It turns out that Chimera 4 also has a damped mobility of the loop by a factor of ~2 compared to AHA (Fig. 6), similarly to PPA. One can also note the reduced mobility of the β8-α8 loop region around residues 310 to 315 in Chimera 4, where the NDW triple mutation was introduced (Fig. 1). Also, the β5-α5 loop (residues 202 to 210) where the Q204L mutation was introduced to enhance packing against the β7-α7 loop is clearly less mobile in Chimera 4. Hence, it appears that our design did achieve a substantial stabilization of the targeted loop and its adjacent contact regions.

## DISCUSSION

The present work shows that it is possible to computationally redesign the temperature dependence of enzyme reactions in a rational way with some knowledge of the key structural determinants. In our case, a specific substrate binding interaction with the universally conserved Asp^264^ had been identified by computer simulations as responsible for the peculiar temperature optimum of the cold-adapted α-amylase (*7*), which occurs about 15°C lower than the melting temperature (*1*). Stabilizing this interaction by redesigning the loop downstream of Asp^264^ and part of its 3D environment indeed turns out to push the *T*-optimum upward, as also predicted by computer simulations of the catalytic reaction. The two designs that were examined experimentally, with 5 and 16 mutations, both show upward shifts of the *T*-optimum. The best predicted design (Chimera 4) has a shift of 6°C and a *k*_cat_ value that is about twice that of the mesophilic PPA enzyme (*20*) but sixfold lower than AHA at 25°C with the CNP-G3 substrate. At 50°C, however, it turns out to be the fastest of the three enzymes.

The mobility of the β7-α7 loop in the Chimera 4 complex with acarbose, as well as in PPA (*7*), is considerably less flexible than in AHA. This reinforces the view that the higher flexibility of key structural regions in cold-adapted enzymes is intimately connected to their higher catalytic efficiency at low temperature (*13*, *27*, *28*). The higher flexibility itself signals a softer protein surface, which is directly reflected by a lower enthalpy penalty for the chemical reaction (*13*, *27*). The back side of the coin, however, appears to be that higher flexibility may cause some interactions to break at temperatures lower than *T*_m_, which in turn can cause a rate optimum that is not related to melting. In this respect, there appears to be a clear trade-off between catalytic efficiency and protein stability. On the other hand, since the working temperature of psychrophilic enzymes is usually much lower than such an optimum, typically near the freezing point of water, this is of little physiological consequence for the organism (*13*).

With regard to the designed Chimera 4, it is also interesting to note that the additional 11 mutations that make it differ from the Loop Graft model are all more than ~13 Å away from the substrate cleavage position. Hence, our results show that the cumulative effect of mutations relatively far away from the enzyme active site can cause substantial changes both in the reaction rate and its temperature dependence. This appears to be a mechanism that has been used by evolution when optimizing enzymes for cold environments. That is, any mutations in an already optimized active site region are likely to be detrimental to catalytic activity, and accordingly, orthologous enzymes from differently adapted species almost never show active site mutations. Hence, adaptation to different environmental temperature regimes is more likely to involve mutations further away from the active site, many of which are actually found on the protein surface (*13*, *28*).

A nice experimental demonstration of the above is a recent laboratory evolution study of the mesophilic *Bacillus subtilis* lipase A (mlipA), which was engineered for higher activity at low temperatures (*29*). On the basis of a population of 16,000 mutants, a variant with five mutations was found that increased the activity sevenfold at 10°C, without any major effect on the temperature optimum. Notably, all five mutations were on the enzyme surface and 11 to 25 Å away from the cleavage site (*29*). These results can be compared to those for the natural psychrophilic lipase A from the arctic bacterium *Bacillus pumilus* (pLipA), which is similarly faster than mLipA (*30*, *31*). The two enzymes differ by only 34 mutations, basically all of which are located on the protein surface, but in that case, pLipA has a *T*-optimum that is 12°C lower than mLipA (*31*). It is thus quite likely that the optimum may tend toward lower temperature in natural evolution, simply due to genetic drift, if the evolutionary pressure mainly acts on the low temperature activity and not on stability at higher temperature (*13*). Such a situation is thus rather different from laboratory evolution where no genetic drift is at play and variants are selected from a pool of mutated mesophilic enzymes. In this context, it is also interesting to note that a recent study of *Escherichia coli* adenylate kinase showed that the introduction of two flexibility-enhancing mutations (Ala→Gly) at positions distal to the reaction site could markedly enhance the activity of the enzyme (*32*). In that case, the enhancement was shown to originate from a more favorable activation entropy for the rate-limiting conformational change associated with product release.

Last, it is interesting to ask whether nature has evolved anything similar to our chimeras. To this end, we searched the National Center for Biotechnology Information nonredundant protein sequence database with the Chimera 4 sequence (residues 200 to 315) as query. It turns out that the highest ranking α-amylase hit, resembling the chimeric sequence more than the WT, is from the recently identified species *Marinimicrobium koreense* with mesophilic characteristics (*33*) (UniProt accession code A0A3N1P4K3). This is a moderately halotolerant bacterium with a reported growth optimum of 35° to 40°C. The enzyme has 59% identity to AHA, and the β7-α7 loop motif sequence is AGGSNVL, compared to AGGSSIL and −GAGNVI for Chimera 4 and AHA, respectively. It is thus more similar to our chimeric models and PPA than to the cold-adapted AHA enzyme in this respect (Fig. 1). The *M. koreense* enzyme also has the Q204L mutation found in Chimera 4 but is more similar to AHA in the three remaining mutation regions (226 to 232, 300, and 310 to 312; Fig. 1B). The Q204L mutation indeed appears to be of particular significance in conjunction with the mesophilic loop motif, as the leucine side chain makes direct contact with the alanine insertion in the PPA crystal structure (Fig. 5A) (*10*). Although the *M. koreense* α-amylase has not been biochemically characterized, it thus appears possible that natural evolution may have been working along the same lines as our computational strategy.

## MATERIALS AND METHODS

### Chimeric models

Crystal structures of AHA (1G94) (*10*) and PPA (1HX0) (*11*) in complex with inhibitors were used as starting points for the calculations. Regions of interest were selected by examining residues near Asp^264^ that are mutated in PPA relative to AHA. The Modeller (*18*) program was used to generate a series of models for each designed sequence. The substrate, ions, and water molecules were taken from the AHA crystal structure. For each designed enzyme variant, 100 models were generated, and from these 100 models, the top six were selected on the basis of the normalized DOPE score (*19*) and manually inspected. If clashes were found, the model was discarded, and the next model with the highest score was added.

### MD simulations

All MD and EVB calculations were carried out with the Q program (*34*, *35*) as described earlier (*7*). Briefly, the different structural models were solvated in a 90-Å-diameter sphere that covers the entire protein. The TIP3P water model (*36*) was used together with the OPLS-AA/M protein force field (*37*). The MD and MD/EVB simulations used the same settings with a 1-fs time step and the local reaction field model (*38*) to handle long-range electrostatics beyond a direct cutoff of 10 Å, except for the reacting groups for which all interactions were explicitly calculated. The temperature was controlled by coupling to a thermal bath with a temperature relaxation time of 10 fs. To determine whether the models preferred the active state or the inactive state of Asp^264^, we carried out six replicate MD simulations at 25°C with different initial conditions for each of the six models of every enzyme variant. For the native AHA enzyme, a single model based on the crystal structure was used to generate 30 independent replicas. The minimized structures were heated to 25°C over 0.15 ns of simulation time with a 10 kcal mol^−1^ Å^−2^ harmonic restraint on all solute heavy atoms. These restraints were then gradually released over 0.15 ns and, after 0.4 ns of unrestrained equilibration, 0.5 ns of data collection followed for each replica. These simulations all used weights of 0.9 and 0.1 for the two EVB states representing reactants and products, which corresponds to the reactant minimum (*7*). RMSF calculations (Fig. 6) for AHA and the crystal structure of Chimera 4 in the reactant state were based on 20 ns of unrestrained MD simulation for each of the structures.

### EVB simulations

MD/EVB simulations for Chimera 4 were run as reported earlier (*7*, *39*) using the free energy perturbation (FEP) umbrella sampling approach (*12*, *13*). The minimized systems were first equilibrated at 25°C, followed by additional equilibration for 50 ps at each target temperature (20°, 25°, 30°, 35°, 40°, and 45°C) with 50% weights of the two EVB states, which approximately corresponds to the transition state of the reaction. After that, the systems were propagated in the forward and backward reaction directions with the FEP method to sample the full reaction path and allow calculations of free energy profiles. The resulting free energy barriers at each temperature are averages over 120 distinct replicas (20 replicas for each of the six structural models of Chimera 4). This corresponds to 186 ns of simulation time for each temperature and a total of 1.1 μs to construct the Arrhenius plot in Fig. 3A.

### Protein production and purification

The gene encoding the AHA α-amylase from *P. haloplanktis*, UniProt accession code P29957, excluding the signal peptides and giving the mature peptide sequence of residues 25 to 477, was codon-optimized for expression in *E. coli* and delivered by GenScript in a pET-22b(+) vector. In addition, two AHA mutants were made, one corresponding to the Loop Graft model with 5 mutations and the other to Chimera 4 with 16 mutations (Fig. 1). These include parts of the porcine PPA sequence with UniProt accession code P00690. The expression vector pET-22b(+) containing WT AHA or mutants was transformed into NiCo21 (DE3) *E. coli* cells (New England Biolabs) by a standard heat shock protocol. ZYP-5052 autoinduction media (1 l) with ampicillin (100 μg/ml) was inoculated with a fresh overnight culture in LB media with ampicillin (100 μg/ml). Cells were grown at 37°C until log phase with an OD_600_ (optical density at 600 nm) of 0.5 to 1.0 before the temperature was lowered to 16°C overnight. Cells were harvested by centrifugation at 6000 rpm for 20 min. The pelleted cells were resuspended in 50 mM Hepes (pH 7.5) with 500 mM NaCl and 10% glycerol before being sonicated with 1-s^−1^ pulses and active cooling for 30 min. The cell supernatant was clarified by centrifugation at 14,000 rpm for 45 min. WT AHA and mutants were affinity-purified using a 5-ml HisTrap HP column (GE Healthcare) in buffer A washed with 5% buffer B [50 mM Hepes (pH 7.5), 500 mM NaCl, 10% glycerol, and 375 mM imidazole], before elution with 5 to 100% buffer B. Fraction peaks were investigated using SDS–12% polyacrylamide gel electrophoresis (PAGE; Bio-Rad). Fractions containing amylase were dialyzed overnight in buffer C [50 mM Hepes (pH 7.5) and 10% glycerol] before anion exchange purification using a 5-ml HiTrap Q column (GE Healthcare) in buffer C. The amylase was eluted with a gradient of buffer D [50 mM Hepes (pH 7.5), 10% glycerol, and 1 M NaCl]. The elution peaks were investigated using SDS-PAGE gel before loading on a HiLoad Superdex 75 16/260 column in phosphate-buffered saline (PBS) buffer.

### Enzyme kinetic assay

Steady-state kinetics measurements were performed at 25°C in 96-well plates (Corning) using a SpectraMax M4 spectrophotometer (Molecular Devices) in PBS buffer. Ninety microliters of 2-chloro-4-nitrophenyl-α-d-maltotrioside substrate dilutions from 5000 to 1.22 μM was mixed with 10-μl enzyme giving a final enzyme concentration in the enzyme assay of 60 nM. The rates were monitored for 30 min, where the initial velocity of hydrolysis against substrate determination was used to determine *k*_cat_ and *K*_M_. Experiments were run in triplicate, and all kinetic data were fitted by nonlinear regression as implemented in the GraphPad Prism software (GraphPad).

### Temperature optimum measurement for activity

Temperature-ramping experiments were performed using the Agilent Cary60 instrument with an accessory Peltier-cooled qChanger6 (Quantum Northwest). In this experiment, 5 mM 2-chloro-4-nitrophenyl-α-d-maltotrioside dissolved in PBS buffer was mixed with the enzyme to a final concentration of 30 nM. The absorbance was followed at 405 nm, and the temperature was ramped with a gradient of 10°C/min. The kinetic data (Fig. 4A) were fitted by numerical integration and optimization functions found in the SciPy package (version 1.5.3) (*40*), with the reaction velocity described by *v*(*T*) = *k*_rxn_(*T*)/[1 + *K*_inact_(*T*)], where *k*_rxn_ represents the rate constant for the chemical step and *K*_inact_ represents the inactivation equilibrium constant. For both of these, the enthalpy and entropy were fitted as free parameters.

### Crystallization conditions for Chimera 4

From a set of eight commercial crystallization screens from Molecular Dimensions, multiple hits were identified for Chimera 4 when screened at 10 mg/ml in the JCSG-plus screen. The screens were set up using the crystallization robot Formulatrix NT8 and the sitting drop method at room temperature. For optimization, a gradient of different pH values of 0.1 M bis-tris and 3.0 M NaCl with the hanging drop method was used to obtain diffraction-quality crystals (pH 5.5 for the apo form and pH 6.0 for the acarbose complex). Prism-shaped crystals appeared after 1.5 to 2 months. One crystal was soaked with 1 mM acarbose to get a structure with an occupied active site. The cryoprotectant was 3.0 M NaCl, 0.1 M bis-tris (pH 5.5 and pH 6.0 for apo and holo forms, respectively), and 30% ethylene glycol. Crystals were flash-frozen in liquid nitrogen after being dipped in the cryoprotectant solution.

### Data collection and structure determination of Chimera 4 and its acarbose complex

X-ray diffraction data extending to 1.7 and 2.05 Å, respectively, were collected at beamlines ID23 and ID30a of the European Synchrotron Radiation Facility, Grenoble, France. Data were processed in X-ray Detector Software (XDS) (*41*). Molecular replacement was performed with Phaser (*42*) using the α-amylase from *P. haloplanktis* with PDB code 1JD7 (*43*) as the search model. Two complete molecules of the maltotetroside-analog acarbose and one molecule containing three of four sugar rings in the acarbose were added to the acarbose-soaked structure, as well as one molecule of acarbose derived pentasaccharide in the active site. Refinement was carried out with phenix.refine (*44*) and iterated with rebuilding in Coot (*45*). Refinement included bulk solvent corrections, individual atomic coordinate, and isotropic B-factor refinement. Riding hydrogens were used during refinement. Solvent molecules were added with phenix.refine and manually curated. Structures were validated using MolProbity (*46*). Data and refinement statistics are given in Table 1. All figures were prepared with PyMOL (version 2.4.1; Schrödinger, LLC).

## Notes

View/request a protocol for this paper from *Bio-protocol*.

## References

[1] S. D’Amico, J. C. Marx, C. Gerday, G. Feller, Activity-stability relationships in extremophilic enzymes. J. Biol. Chem. 278, 7891–7896 (2003). 12511577 10.1074/jbc.M212508200

[2] D. Georlette, B. Damien, V. Blaise, E. Depiereux, V. N. Uversky, C. Gerday, G. Feller, Structural and functional adaptations to extreme temperatures in psychrophilic, mesophilic, and thermophilic DNA ligases. J. Biol. Chem. 278, 37015–37023 (2003). 12857762 10.1074/jbc.M305142200

[3] T. Collins, M. A. Meuwis, C. Gerday, G. Feller, Activity, stability and flexibility in glycosidases adapted to extreme thermal environments. J. Mol. Biol. 328, 419–428 (2003). 12691750 10.1016/s0022-2836(03)00287-0

[4] R. M. Daniel, M. J. Danson, A new understanding of how temperature affects the catalytic activity of enzymes. Trends Biochem. Sci. 35, 584–591 (2010). 20554446 10.1016/j.tibs.2010.05.001

[5] J. K. Hobbs, W. Jiao, A. D. Easter, E. J. Parker, L. A. Schipper, V. L. Arcus, Change in heat capacity for enzyme catalysis determines temperature dependence of enzyme catalyzed rates. ACS Chem. Biol. 8, 2388–2393 (2013). 24015933 10.1021/cb4005029

[6] V. Nguyen, C. Wilson, M. Hoemberger, J. B. Stiller, R. V. Agafonov, S. Kutter, J. English, D. L. Theobald, D. Kern, Evolutionary drivers of thermoadaptation in enzyme catalysis. Science 355, 289–294 (2017). 28008087 10.1126/science.aah3717 PMC5649376

[7] J. Socan, M. Purg, J. Åqvist, Computer simulations explain the anomalous temperature optimum in a cold-adapted enzyme. Nat. Commun. 11, 2644 (2020). 32457471 10.1038/s41467-020-16341-2 PMC7250929

[8] J. Åqvist, J. Socan, M. Purg, Hidden conformational states and strange temperature optima in enzyme catalysis. Biochemistry 59, 3844–3855 (2020). 32975950 10.1021/acs.biochem.0c00705 PMC7584337

[9] J. Åqvist, F. van der Ent, Calculation of heat capacity changes in enzyme catalysis and ligand binding. J. Chem. Theor. Comput. 18, 6345–6353 (2022). 10.1021/acs.jctc.2c00646 PMC9558309 36094903

[10] N. Aghajari, M. Roth, R. Haser, Crystallographic evidence of a transglycosylation reaction: Ternary complexes of a psychrophilic α-amylase. Biochemistry 41, 4273–4280 (2002). 11914073 10.1021/bi0160516

[11] M. Qian, V. Nahourn, J. Bonicel, H. Bischoff, B. Henrissat, F. Payan, Enzyme-catalyzed condensation reaction in a mammalian α-amylase. high-resolution structural analysis of an enzyme−Inhibitor complex. Biochemistry 40, 7700–7709 (2001). 11412124 10.1021/bi0102050

[12] J. Åqvist, A. Warshel, Simulation of enzyme reactions using valence bond force fields and other hybrid quantum/classical approaches. Chem. Rev. 93, 2523–2544 (1993).

[13] J. Åqvist, G. V. Isaksen, B. O. Brandsdal, Computation of enzyme cold adaptation. Nat. Rev. Chem. 1, 0051 (2017).

[14] S. Janecek, α-Amylase family: Molecular biology and evolution. Prog. Biophys. Mol. Biol. 67, 67–97 (1997). 9401418 10.1016/s0079-6107(97)00015-1

[15] E. A. MacGregor, S. Janecek, B. Svensson, Relationship of sequence and structure to specificity in the α-amylase family of enzymes. Biochim. Biophys. Acta 1564, 1–20 (2001). 10.1016/s0167-4838(00)00302-2 11257505

[16] S. M. Malakauskas, S. L. Mayo, Design, structure and stability of a hyperthermophilic protein variant. Nat. Struct. Biol. 5, 470–475 (1998). 9628485 10.1038/nsb0698-470

[17] S. D’Amico, C. Gerday, G. Feller, Temperature adaptation of proteins: Engineering mesophilic-like activity and stability in a cold-adapted α-amylase. J. Mol. Biol. 332, 981–988 (2003). 14499602 10.1016/j.jmb.2003.07.014

[18] A. Šali, T. L. Blundell, Comparative protein modelling by satisfaction of spatial restraints. J. Mol. Biol. 234, 779–815 (1993). 8254673 10.1006/jmbi.1993.1626

[19] M. Shen, A. Šali, Statistical potential for assessment and prediction of protein structures. Prot. Sci. 15, 2507–2524 (2006). 10.1110/ps.062416606 PMC2242414 17075131

[20] B. A. Lund, B. O. Brandsdal, ThermoSlope: A software for determining thermodynamic parameters from single steady-state experiments. Molecules 26, 7155 (2021). 34885737 10.3390/molecules26237155 PMC8658824

[21] C. Li, A. Begum, S. Numao, K. H. Park, S. G. Withers, G. D. Brayer, Acarbose rearrangement mechanism implied by the kinetic and structural analysis of human pancreatic α-amylase in complex with Analogues and their elongated counterparts. Biochemistry 44, 3347–3357 (2005). 15736945 10.1021/bi048334e

[22] F. Payan, M. Qian, Crystal structure of the pig pancreatic α-amylase complexed with malto-oligosaccharides. J. Prot. Chem. 22, 275–284 (2003). 10.1023/a:1025072520607 12962327

[23] M. Qian, S. Spinelli, H. Driguez, F. Payan, Structure of a pancreatic α-amylase bound to substrate analogue at 2.03 Å resolution. Prot. Sci. 6, 2285–2296 (1997). 10.1002/pro.5560061102 PMC2143580 9385631

[24] M. Machius, L. Vertsesy, R. Huber, G. Wiegand, Carbohydrate and protein-based inhibitors of porcine pancreatic α-amylase: Structure analysis and comparison of their binding characteristics. J. Mol. Biol. 260, 409–421 (1996). 8757803 10.1006/jmbi.1996.0410

[25] N. Aghajari, G. Feller, C. Gerday, R. Haser, Crystal structures of the psychrophilic α-amylase from *Alteromonas haloplanctis* in its native form and complexed with an inhibitor. Prot. Sci. 7, 564–572 (1998). 10.1002/pro.5560070304 PMC2143949 9541387

[26] R. Maurus, A. Begum, L. K. Williams, J. R. Fredriksen, R. Zhang, S. G. Withers, G. D. Brayer, Alternative catalytic anions differentially modulate human α-amylase activity and specificity. Biochemistry 47, 3332–3344 (2008). 18284212 10.1021/bi701652t

[27] G. V. Isaksen, J. Åqvist, B. O. Brandsdal, Enzyme surface rigidity tunes the temperature dependence of catalytic rates. Proc. Natl. Acad. Sci. U.S.A. 113, 7822–7827 (2016). 27354533 10.1073/pnas.1605237113 PMC4948340

[28] J. Sǒan, G. V. Isaksen, B. O. Brandsdal, J. Åqvist, Towards rational computational engineering of psychrophilic enzymes. Sci. Rep. 9, 19147 (2019). 31844096 10.1038/s41598-019-55697-4 PMC6915740

[29] V. Kumar, P. Yedavalli, V. Gupta, N. M. Rao, Engineering lipase A from mesophilic *Bacillus subtilis* for activity at low temperatures. Prot. Eng. Des. Selec. 27, 73–82 (2014). 10.1093/protein/gzt064 24402332

[30] A. R. Wi, S. J. Jeon, S. Kim, H. J. Park, D. Kim, S. J. Han, J. H. Yim, H. W. Kim, Characterization and a point mutational approach of a psychrophilic lipase from an arctic bacterium, Bacillus pumilus. Biotechnol. Lett. 36, 1295–1302 (2014). 24563306 10.1007/s10529-014-1475-8

[31] F. van der Ent, B. A. Lund, L. Svalberg, M. Purg, G. Chukwu, M. Widersten, G. V. Isaksen, B. O. Brandsdal, J. Åqvist, Structure and mechanism of a cold-adapted bacterial lipase. Biochemistry 61, 933–942 (2022). 35503728 10.1021/acs.biochem.2c00087 PMC9118546

[32] H. G. Saavedra, J. O. Wrabl, J. A. Anderson, J. Li, V. J. Hilser, Dynamic allostery can drive cold adaptation in enzymes. Nature 558, 324–328 (2018). 29875414 10.1038/s41586-018-0183-2 PMC6033628

[33] J. M. Lim, C. O. Jeon, J. C. Lee, S. M. Song, K. Y. Kim, C. J. Kim, Marinimicrobium koreense gen. nov., sp. nov. and Marinimicrobium agarilyticum sp. nov., novel moderately halotolerant bacteria isolated from tidal flat sediment in Korea. Int. J. Syst. Evol. Microbiol. 56, 653–657 (2006). 16514044 10.1099/ijs.0.64075-0

[34] J. Marelius, K. Kolmodin, I. Feierberg, J. Åqvist, Q: A molecular dynamics program for free energy calculations and empirical valence bond simulations in biomolecular systems. J. Mol. Graph. Model. 16, 213–225 (1998). 10522241 10.1016/s1093-3263(98)80006-5

[35] P. Bauer, A. Barrozo, M. Purg, B. A. Amrein, M. Esguerra, P. B. Wilson, D. T. Major, J. Åqvist, S. C. L. Kamerlin, Q6: A comprehensive toolkit for empirical valence bond and related free energy calculations. SoftwareX 7, 388–395 (2018).

[36] W. L. Jorgensen, J. Chandrasekhar, J. D. Madura, R. W. Impey, M. L. Klein, Comparison of simple potential functions for simulating liquid water. J. Chem. Phys. 79, 926–935 (1983).

[37] M. J. Robertson, J. Tirado-Rives, W. L. Jorgensen, Improved peptide and protein torsional energetics with the OPLS-AA force field. J. Chem. Theory Comput. 11, 3499–3509 (2015). 26190950 10.1021/acs.jctc.5b00356 PMC4504185

[38] F. S. Lee, A. Warshel, A local reaction field method for fast evaluation of long-range electrostatic interactions in molecular simulations. J. Chem. Phys. 97, 3100–3107 (1992).

[39] L. Koenekoop, F. van der Ent, M. Purg, J. Åqvist, The activation parameters of a cold-adapted short chain dehydrogenase are insensitive to enzyme oligomerization. Biochemistry 61, 514–522 (2022). 35229609 10.1021/acs.biochem.2c00024 PMC8988307

[40] P. Virtanen, R. Gommers, T. E. Oliphant, M. Haberland, T. Reddy, D. Cournapeau, E. Burovski, P. Peterson, W. Weckesser, J. Bright, S. J. van der Walt, M. Brett, J. Wilson, K. J. Millman, N. Mayorov, A. R. J. Nelson, E. Jones, R. Kern, E. Larson, C. J. Carey, İ. Polat, Y. Feng, E. W. Moore, J. V. Plas, D. Laxalde, J. Perktold, R. Cimrman, I. Henriksen, E. A. Quintero, C. R. Harris, A. M. Archibald, A. H. Ribeiro, F. Pedregosa, P. van Mulbregt; SciPy 1.0 contributors, SciPy 1.0: Fundamental algorithms for scientific computing in Python. Nat. Methods 17, 261–272 (2020). 32015543 10.1038/s41592-019-0686-2 PMC7056644

[41] W. Kabsch, XDS. Acta Cryst. 66, 125–132 (2010). 10.1107/S0907444909047337 PMC2815665 20124692

[42] A. J. McCoy, R. W. Grosse-Kunstleve, P. D. Adams, M. D. Winn, L. C. Storoni, R. J. Read, Phaser crystallographic software. J. Appl. Cryst. 40, 658–674 (2007). 19461840 10.1107/S0021889807021206 PMC2483472

[43] N. Aghajari, G. Feller, C. Gerday, R. Haser, Structural basis of α-amylase activation by chloride. Prot. Sci. 11, 1435–1441 (2002). 10.1110/ps.0202602 PMC2373612 12021442

[44] P. V. Afonine, R. W. Grosse-Kunstleve, N. Echols, J. J. Headd, N. W. Moriarty, M. Mustyakimov, T. C. Terwilliger, A. Urzhumtsev, P. H. Zwart, P. D. Adams, Towards automated crystallographic structure refinement withphenix.refine. Acta Crystallogr. 68, 352–367 (2012). 10.1107/S0907444912001308 PMC3322595 22505256

[45] P. Emsley, Coot: Model-building tools for molecular graphics. Acta Crystallogr. 60, 2126–2132 (2004). 10.1107/S0907444904019158 15572765

[46] I. W. Davis, A. Leaver-Fay, V. B. Chen, J. N. Block, G. J. Kapral, X. Wang, L. W. Murray, W. B. Arendall III, J. Snoeyink, J. S. Richardson, D. C. Richardson, MolProbity: All-atom contacts and structure validation for proteins and nucleic acids. Nucl. Acids Res. 35, W375–W383 (2007). 17452350 10.1093/nar/gkm216 PMC1933162

