# Supplementary material for: Computational design of the temperature optimum of an enzyme reaction
Source: Sci Adv. 2023 Jun 28;9(26):eadi0963. doi: 10.1126/sciadv.adi0963 (PMC10306287; doi:10.1126/sciadv.adi0963)
Supplement: Supplementary file 1 — Fig. S1 Table S1 [file sciadv.adi0963_sm.pdf]

Science Advances
AAAS

Supplementary Materials for

Computational design of the temperature optimum of an enzyme reaction

Florian van der Ent *et al.*

Corresponding author: Johan Åqvist, aqvist@xray.bmc.uu.se

Sci. Adv. **9**, eadi0963 (2023)
DOI: 10.1126/sciadv.adi0963

This PDF file includes:

Fig. S1
Table S1

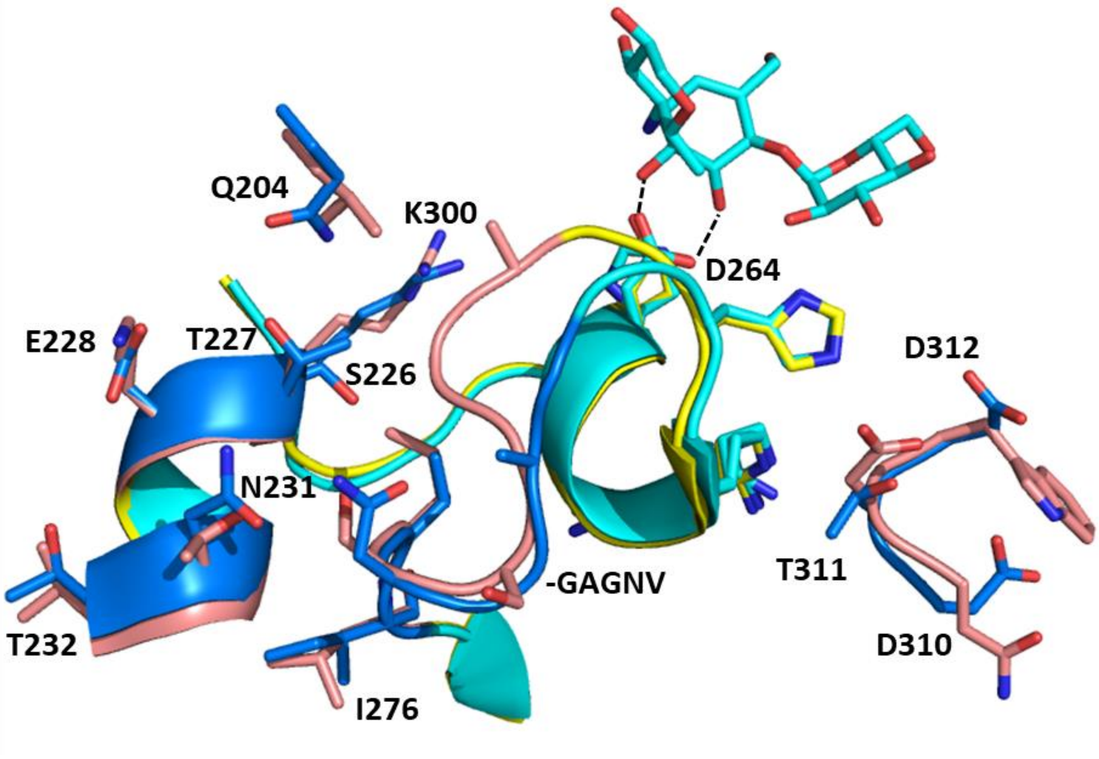

Fig. S1.
Mutated regions in the chimeric variants. Besides the –GAGNV/AGGSSI mutations in the Loop Graft variant, a set of additional positions (AHA numbering) were selected for substitution in the other chimeras. These positions interact with the β7-α7 loop and were considered to possibly stabilize the loop conformation. The AHA crystal structure (1G94) (*10*) is shown in cyan with mutated residues in blue and the PPA structure (1HX0) (*11*) is shown in yellow with the target residues for mutation in pink. The key interaction between Asp264 and the substrate is also indicated.

Table S1.
Kinetic parameters from steady-state measurements at 25°C with the CNP-G3 substrate.

	K_M (mM)	k_{cat} (min^{-1})	k_{cat}/K_M (M^{-1} min^{-1})
**AHA wt**	2.81 ± 0.11	106.5 ± 6.6	37722
**Loop Graft**	2.24 ± 0.22	73.0 ± 3.2	32589
**Chimera 4**	3.49 ± 0.35	17.7 ± 1.0	5072
